# A Novel Serum Biomarker Model to Discriminate Aortic Dissection from Coronary Artery Disease

**DOI:** 10.1155/2022/9716424

**Published:** 2022-07-20

**Authors:** Peijiang Lu, Xin Feng, Rui Li, Peng Deng, Shiliang Li, Jiewen Xiao, Jing Fang, Xingyu Wang, Chang Liu, Qiuxia Zhu, Jing Wang, Zemin Fang, Lu Gao, Sen Guo, Xue-Jun Jiang, Xue-Hai Zhu, Tingting Qin, Xiang Wei, Xin Yi, Ding-Sheng Jiang

**Affiliations:** ^1^Division of Cardiothoracic and Vascular Surgery, Sino-Swiss Heart-Lung Transplantation Institute, Tongji Hospital, Tongji Medical College, Huazhong University of Science and Technology, Wuhan, Hubei, China; ^2^Key Laboratory of Organ Transplantation, Ministry of Education; NHC Key Laboratory of Organ Transplantation; Key Laboratory of Organ Transplantation, Chinese Academy of Medical Sciences, Wuhan, Hubei, China; ^3^Department of Cardiology, Renmin Hospital of Wuhan University, Wuhan, Hubei, China; ^4^Department of Cardiology, The First Affiliated Hospital of Zhengzhou University, Zhengzhou, Henan, China; ^5^Department of Biliary-Pancreatic Surgery, Tongji Hospital, Tongji Medical College, Huazhong University of Science and Technology, Wuhan, Hubei, China

## Abstract

**Background:**

The misdiagnosis of aortic dissection (AD) can lead to a catastrophic prognosis. There is currently a lack of stable serological indicators with excellent efficacy for the differential diagnosis of AD and coronary artery disease (CAD). A recent study has shown an association between AD and iron metabolism. Thus, we investigated whether iron metabolism could discriminate AD from CAD.

**Methods:**

This retrospective and multicenter cross-sectional study investigated the efficacy of biomarkers of iron metabolism for the differential diagnosis of AD. We collected biomarkers of iron metabolism, liver function, kidney function, and other biochemistry test, and further, logistic regression analysis was applied.

**Results:**

Between Oct. 8, 2020, and Mar. 1, 2021, we recruited 521 patients diagnosed with AD, CAD, and other cardiovascular diseases (OCDs) with the main symptoms of chest and back pain and assigned them to discovery set (*n* = 330) or validation set (*n* = 191). We found that six serum biomarkers, including serum iron, low-density lipoprotein, uric acid, transferrin, high-density lipoprotein, and estimated glomerular filtration rate, can serve as a novel comprehensive indicator (named FLUTHE) for the differential diagnosis of AD and CAD with a sensitivity of 0.954 and specificity of 0.905 to differentially diagnose AD and CAD more than 72 h past symptom onset.

**Conclusion:**

Our findings provide insight into the role of iron metabolism in diagnosing and distinguishing AD, which might in the future be a key component in AD diagnosis. Furthermore, we establish a novel model named “FLUTHE” with higher efficiency, safety, and economy, especially for patients with chest pain for more than 72 h.

## 1. Introduction

Aortic dissection (AD) is a fatal emergency occurring after a tear in the aortic intima or bleeding within the aortic wall, which results in the dissection of layers in the aortic wall [1]. AD is one of the most common acute aortic conditions, with an incidence of 6 cases every 100,000 people per year, which may be underestimated for not accounting for preadmission deaths [2]. A prospective analysis of 30,412 middle-aged men and women with acute aortic dissection (AAD) with 20 years of follow-up reported 15 cases every 100,000 people per year at risk for AD. Moreover, among those 65-75 years of age, the incidence can even reach 35 cases every 100,000 people [3]. The mortality increases by 1%~2% per hour after symptom onset, for which the cumulative mortality will finally reach 27.4% overall and even reach 58% in those with type A dissection, who are not operated on [4]. Since the main symptom of AD is chest and back pain, AD should be distinguished from other sudden-onset severe chest and back pain diseases, especially acute myocardial infarction (AMI) [1]. Because the treatments of these diseases were different [5], developing a method that provides a rapid and early indication to diagnose AD and exclude other diseases is crucial for improving the treatment rate of AD.

At present, numbers of imaging examinations have been applied to diagnose AD, including X-ray, computed tomography (CT)/CT angiography (CTA), and magnetic resonance imaging (MRI)/MRI angiography (MRA) [6]. Before the patient undergoes CTA examination, more evidence is needed to reduce unnecessary examinations and risks. Many researchers have focused on serum biomarkers for years and developed a few to diagnose AD, such as those associated with vascular interstitium (calponin), interleukin (IL)-1 receptor family member (sST2), and fibrinolytic function (D-dimer) [7–9]. Among these biomarkers, D-dimer is one of the most common clinical indicators with a sensitivity and negative likelihood ratio of 98.0% (95% CI: 96.3% to 99.1%) and 0.05 (95% CI: 0.03 to 0.09), respectively, but with relatively low specificity [8–11]. It has been reported that patients with AD had an accompanying elevation in D-dimer, which was 5- to 10-fold greater than that in control subjects in the initial 24 h [7], but it decreased after 24 h from the onset time. This feature limits the specificity and time window of D-dimer in the diagnosis. Therefore, the development of more specific, sensitive, and stable serum biomarkers to diagnose AD has great clinical significance.

Iron, a vital element in all living things and involved in nearly all metabolic reactions, is often used for diagnosis [12–14]. Recent research has found low iron levels in patients with heart failure, which results in poor prognosis [15]. In addition, CAD is suggested to occur with frequent iron deficiency that also predicts a higher likelihood of death, particularly in patients with high-risk profiles [16]. Moreover, a small sample study revealed that the iron level was elevated in aortic tissues of AD patients compared with the control group, which indicated the potential importance of iron in the pathological processes of AD and may serve as a promising diagnostic indicator [17]. However, whether biomarkers of iron metabolism can be reliable predictors for AD remains unknown.

## 2. Methods

### 2.1. Study Samples

We collected data from 521 patients from three clinical centers (Tongji Hospital, Renmin Hospital of Wuhan University, and the First Affiliated Hospital of Zhengzhou University). The study was approved by the Ethics Review Board of Tongji Hospital, Tongji Medical College, Huazhong University of Science and Technology. We enrolled the patients diagnosed with aortic dissection, coronary artery disease, or other cardiovascular diseases according to the diagnosis criteria as mentioned in Supplemental Methods in any of the three hospitals from Oct. 8, 2020, to Mar. 1, 2021, and the patients should include complete clinical information, including iron metabolism-related biomarkers, blood routine, blood chemistry, liver and kidney function tests, D-dimer, CT/CTA, MRI/MRA, and ECG. The patients (a) who received packed red blood cells, whole blood, or platelets less than 10 days before the blood sample were taken; (b) the patients with aortic trauma, pseudoaneurysm, a history of anemia or obvious anemia symptoms or other hemopathy, a history of heart failure, a history of acute/chronic intestinal diseases, a history of chronic renal dysfunction, severe pulmonary diseases, or active cancer and (c) the patients who received surgery before the blood sample was taken were excluded.

To evaluate the diagnostic performance of biomarkers of iron metabolism in discriminating AD from other non-AD cardiovascular diseases, we firstly established a retrospective, frequency-matched, case-control study set. Considering the urgent need for treatment, we divided all patients with AD into different time intervals according to the time from symptom onset to blood sample collection. AD patients within the same time frame were mainly selected from Tongji hospitals. For the control groups, the patients were selected from all three hospitals. The whole design is shown in Table 1.

As a result, the patients were divided into 2 study sets according to the time of admission, namely, discovery set and validation set. Patients admitted from Oct. 8, 2020, to Jan. 8, 2021, were assigned to the discovery set, and the others were assigned to the validation set (Jan. 9, 2021, to Mar. 1, 2021). As a result, the discovery set included 330 patients (162 patients with AD, 135 patients with CAD, and 33 with OCDs), and the validation cohort set included 191 patients (105 patients with AD and 86 patients with non-AD diseases).

### 2.2. Measurements of Biomarkers Associated with Iron Metabolism and Other Markers

Measurements of biomarkers associated with iron metabolism, D-dimer, liver functions, kidney functions, and blood chemistry were performed, including serum iron (sFe), transferrin (TF), total iron-binding capacity (TIBC), transferrin saturation (TFS), ferritin, unsaturated iron-binding capacity (UIBC), soluble transferrin receptor (sTFR), uric acid (UA), estimated glomerular filtration rate (eGFR), low-density lipoprotein (LDL), and high-density lipoprotein (HDL). The further information on measurements of iron metabolism and other markers was shown in Supplemental Methods.

### 2.3. Statistical Analysis

Demographic and medical information of AD or non-AD diseases were summarized by the mean (95% CI) for normal variables (e.g., TF). An independent sample *t*-test was used to compare mean levels of log-transformed D-dimer or other continuous risk factors (log-transformed where appropriate) by different disease outcomes. One-way ANOVA was used to compare the mean levels of continuous risk factors, which were divided into more than 2 groups. The *χ*2 test was used to assess differences in the distribution of categorical variables by different disease outcomes. The further information on statistical analysis of model establishment and evaluation was shown in Supplemental Methods.

## 3. Results

### 3.1. Patient Demographics and Biomarkers of Iron Metabolism Distribution

**Figure 1:**
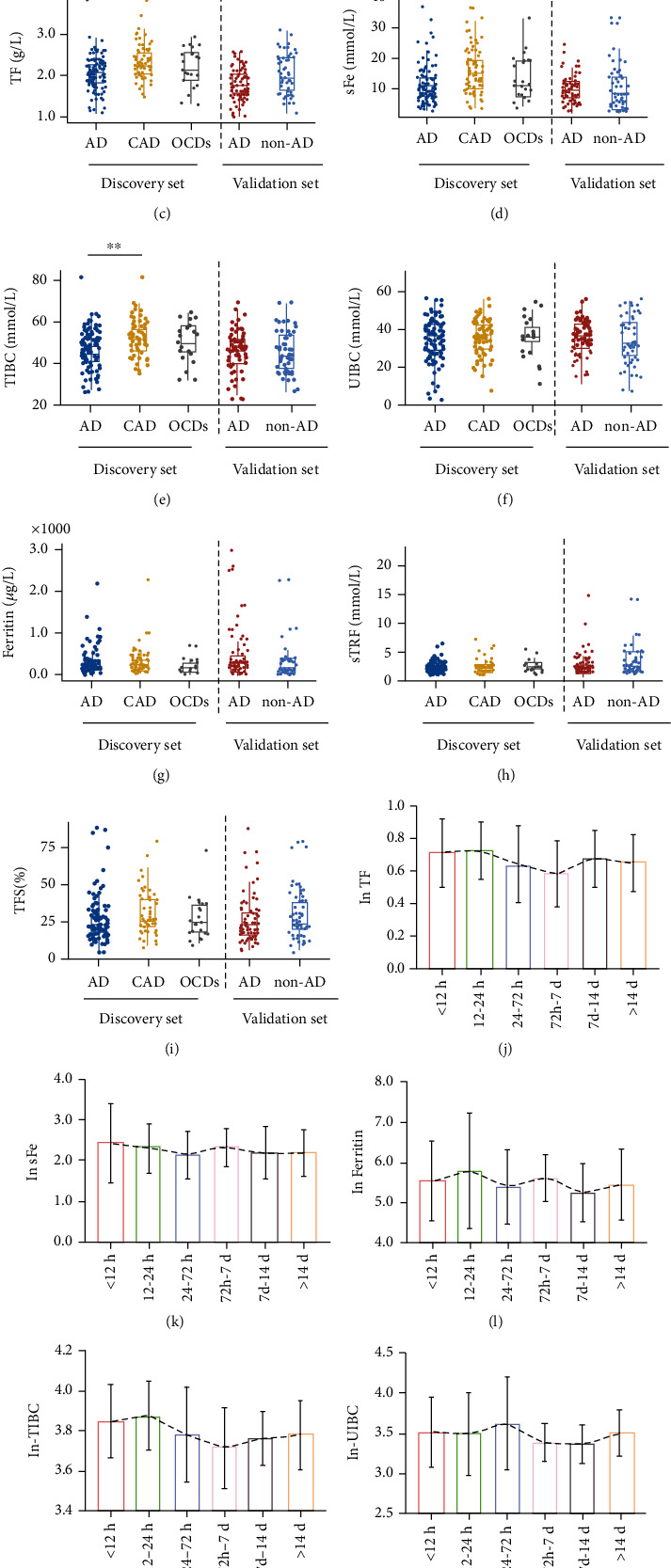
Distributions of biomarkers according to disease status and the time from symptom onset in AD patients. (a) Distributions of D-dimer according to disease status; (b) distributions of D-dimer according to the time from symptom onset in AD patients processed by the Napierian logarithm; (c–i) distributions of biomarkers associated with iron metabolism according to disease status (c) TF, (d) sFe, (e) TIBC, (f) UIBC, (g) ferritin, (h) sTFR, and (i) TFS; (j–p) distributions of biomarkers associated with iron metabolism according to the time from symptom onset in AD patients processed by the Napierian logarithm: (j) TF, (k) sFe, (l) ferritin, (m) TIBC, (n) UIBC, (o) sTFR, and (p) TFS. ∗*P* < 0.05; ∗∗*P* < 0.01; ∗∗∗*P* < 0.001; ∗∗∗∗*P* < 0.0001. AD: aortic dissection; CAD: coronary artery disease; sFe: serum iron; TF: transferrin; TIBC: total iron-binding capacity; UIBC: unsaturated iron-binding capacity; sTFR: soluble transferrin receptor; TFS: transferrin saturation.

To investigate the diagnostic effectiveness of biomarkers associated with iron metabolism in patients with AD, this cross-sectional study was assigned into a discovery set and a validation set divided according to the time point Jan. 8, 2021 (Table 1). In the discovery set, the baseline characteristics of the patients are shown in Supplemental Table S1 and S2. Similar as in clinical practice, in the present study, compared with CAD patients, D-dimer was increased in AD patients in both the discovery set and validation set (Figure 1(a) and Supplemental Table S1, S2). It was reported that the level of D-dimer would decline rapidly after the first 24 h from symptom onset, which may account for D-dimer's performance loss in time distribution when diagnosing AD. In our study, of the 162 patients who underwent dissection, the peak level of D-dimer occurred within 24 h of symptoms (Figure 1(b)), which provided supportive evidence for the reference guideline.

Thus, to find a more stable indicator with excellent diagnostic effectiveness, we firstly detected the serum levels of biomarkers of iron metabolism. We found that the serum levels of TF and sFe were lower in AD patients than in CAD patients (Figures 1(c) and 1(d)). However, comparable contents of UIBC, TIBC, ferritin, sTFR, and TFS were detected in patients in the indicated groups (Figures 1(e)–1(i)). More importantly, the serum content of the biomarkers of iron metabolism remained relatively stable at different times after the onset of symptoms of AD (Figures 1(j)–1(p)). This indicated that biomarkers of iron metabolism could serve as potential indicators to diagnose AD at different time after symptom onset.

What surprised us was that nearly half patients diagnosed with AD patients were accompanied by lower level of sFe and transferrin (Table 2). This suggested an abnormality in iron metabolism of patients with aortic dissection and a potential diagnosis value for AD.

### 3.2. FLUTHE Showed More Powerful Performance than a Single Biomarker in ROC Analyses

**Figure 2:**
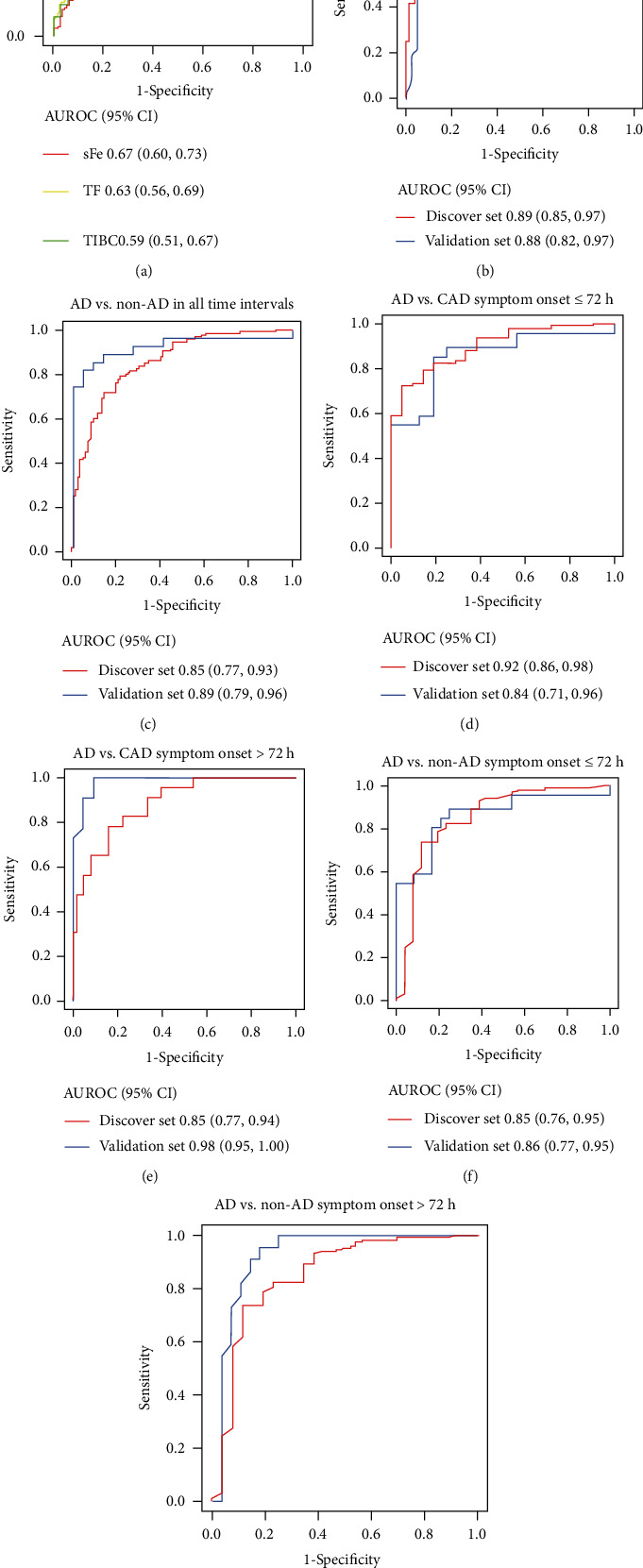
Receiver operating characteristic curves for biomarkers according to the time from symptom onset. (a) Single factor between AD and CAD in the discovery set. (b) FLUTHE between AD and CAD patients of the discovery set in all-time intervals. (c) FLUTHE between AD and non-AD disease patients of the discovery set in all-time intervals. (d) FLUTHE between AD and CAD patients in the discovery set within the first 72 h from symptom onset. (e) FLUTHE between AD and CAD patients in the discovery set over the first 72 h from symptom onset. (f) FLUTHE between AD and non-AD patients in the discovery set within the first 72 h from symptom onset. (g) FLUTHE between AD and non-AD patients in the discovery set over the first 72 h from symptom onset. AD: aortic dissection; CAD: coronary artery disease; sFe: serum iron; TF: transferrin; TIBC: total iron-binding capacity.

To further investigate the potential diagnostic efficiency of sFe and TF, ROC analyses were performed to determine the diagnostic performance. In the discovery set, the AUROCs for 162 patients with AD versus 135 patients with CAD for sFe, TF, and TIBC were 0.67 (95% CI: 0.60, 0.73), 0.63 (95% CI: 0.56, 0.69), and 0.59 (95% CI: 0.51, 0.87), respectively (Figure 2(a)). Thus, to improve the diagnostic performance of biomarkers associated with iron metabolism, logistic regression was performed to create models with iron metabolism biomarkers for discriminating AD from non-AD, especially CAD. Given that only two covariates of iron metabolism were found to have a significant association with outcome, sFe (*P* = 0.006) and TF (*P* = 0.002) (Supplemental Table S3), other serum examinations were performed, including liver function, kidney function, and blood biochemistry tests, to further improve the diagnostic performance. We found that four biomarkers were significantly related to the outcome of highest performance: UA (*P* = 0.022), eGFR (*P* < 0.0001), LDL (*P* < 0.0001), and HDL (*P* = 0.009) (Supplemental Table S3). As a result, sFe, LDL, UA, TF, HDL, and eGFR were incorporated as a prediction combination (FLUTHE). The formula for calculating FLUTHE is shown as

(1) $$\begin{array}{c} Z=\exp\left(4.831-0.067\left[\text{sFe }\left(\mu \text{mol}/\text{mL}\right)\right]+0.854\left[\text{LDL}\left(\text{mmol}/\text{mL}\right)\right]-0.003\left[\text{UA}\left(\mu \text{mol}/\text{L}\right)\right]-1.382\left[\text{TF}\left(\text{g}/\text{mL}\right)\right]+1.674\left[\text{HDL}\left(\text{mmol}/\text{mL}\right)\right]-0.037\left[\text{eGFR}\left(\frac{\text{ml}/\min}{1.73{\text{m}}^{2}}\right)\right]\right., \end{array}$$

(2) $$\begin{array}{c} \text{FLUTHE}=\frac{Z}{1+Z}. \end{array}$$

The results of ROC analyses showed that FLUTHE exhibited an obvious advantage for discriminating AD from CAD and non-AD diseases compared with sFe and TF in the discovery set (Figures 2(b) and 2(c)).

Patients were grouped according to whether symptom onset was more than 72 h past. In the discovery set, FLUTHE had a comparable diagnostic power in different time distributions for differentiating AD group from either CAD or non-AD group (Figures 2(d)–2(g)).

### 3.3. Sensitivity Analysis of FLUTHE Showed a Relative Stable Performance within or over 72 h after Symptom Onset

In the discovery set, FLUTHE at a cutoff level of 0.648 led to the maximum summation of sensitivity and specificity in discriminating AD from non-AD diseases. In general, FLUTHE showed a high specificity in both groups where AD patients were compared with both patients with CAD (0.917) and patient with non-AD (0.854) in discovery set. The positive predictive value was over 0.90 in the subgroups versus CAD, which indicated that FLUTHE was an ideal rule-out tool to discriminate AD from non-AD diseases, especially to discriminate AD from CAD (Supplemental Table S4).

Within the first 72 h from symptom onset, FLUTHE was a promising indicator for differentiating AD from CAD. While over 72 h, FLUTHE showed a good performance in specificity (Table 3). For differentiating AD from non-AD patients, the results were similar to those from CAD (Supplemental Table S5).

Together, these results veiled that FLUTHE could be a novel model to discriminate AD from CAD. More importantly, FLUTHE showed a relative stable diagnostic value in different time distributions, especially in specificity.

### 3.4. Verification of the Findings in the Validation Set

To verify the diagnostic effectiveness of FLUTHE, an independent validation set was built. The baseline characteristics of the patients are shown in Supplemental Table S2. The content of D-dimer in AD patients was higher, while TF was lower than those in non-AD patients (Figure 1(a)). Other biomarkers of iron metabolism were comparable between AD patients and non-AD patients.

Unsurprisingly, the results of ROC and sensitivity analysis in the validation set showed that FLUTHE was a good indicator for the differential diagnosis of AD and CAD or non-AD (Figures 2(b) and 2(c)). Compared to the results in the discovery set, in the first 72 h from symptom onset, FLUTHE showed a slightly lower performance in the validation set to the differentially diagnose AD and CAD or non-AD (Figures 2(d) and 2(f)). However, after 72 h of symptom onset, FLUTHE showed a more powerful performance for discriminating AD from CAD or non-AD (Figures 2(e) and 2(g)). Sensitivity analysis also demonstrated that, consistent with the results in the discovery set, FLUTHE showed high specificity in general (Table 3, Supplemental Table S4 and Supplemental Table S5).

### 3.5. FLUTHE Score System for AD Diagnosis Derivation from Model FLUTHE

**Figure 3:**
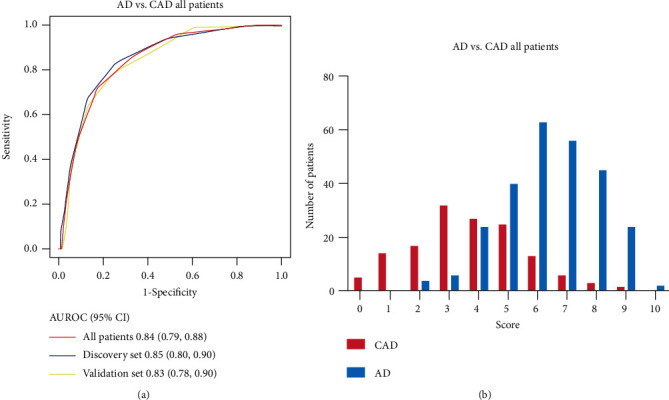
Receiver operating characteristic curves of biomarkers according to the time from symptom onset. (a) FLUTHE score system between AD and CAD patients in all-time intervals. (b) The distribution of FLUTHE score system between AD and CAD patients. AD: aortic dissection; CAD: coronary artery disease.

For further clinical practice, we simplify the model FLUTHE into a score system to assistant AD diagnosis. Six variables were identified as risk factors: higher level of HDL and LDL, lower level of sFe, transferrin, eGFR, and UA. These 6 factors were incorporated into the diagnosis prediction score using the integer point values from the *β* coefficient and reference value of each variable as described; also to emphasize the transferrin, it was assigned with more scores (Supplemental Table S3). The score system ranged from 0 to 10 points. ROC curves are shown in Figure 3(a), and the distribution of different scores were shown in Figure 3(b). The whole score system was divided into 3 level of risk: low probability group (score, 0-3), medium probability group (score, 4-5), and high probability group (score, 6-10). The low probability group demonstrated lower possibility of AD than the medium probability group and high probability group (Table 4).

**Figure 4:**
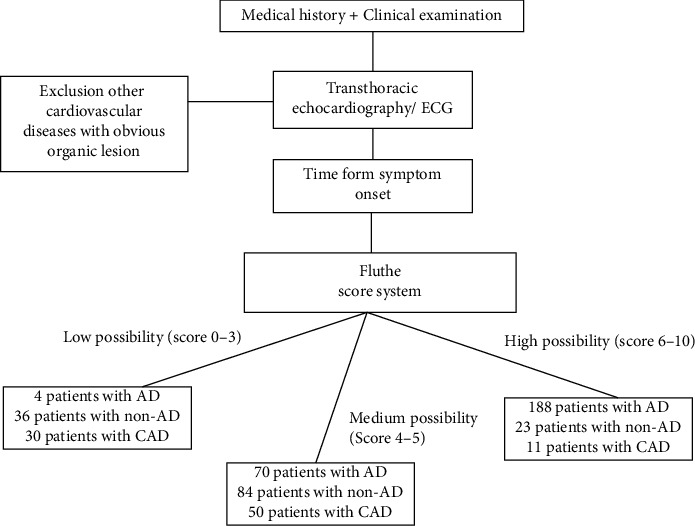
Flowchart summarizing the diagnostic workup. For patients in the discovery and validation sets with FLUTHE score system, low possibility group includes 4 patients with AD and 36 patients with non-AD (including 30 with CAD). High possibility group includes 188 patients with AD and 23 patients with non-AD (including 11 with CAD). Medium possibility group includes 70 patients with AD and 84 patients with non-AD (including 50 with CAD). AD: aortic dissection; CAD: coronary artery disease.

Overall, FLUTHE score system results were available in 405 (77.7%) patients. A diagnostic flowchart including both patients is presented in Figure 4.

## 4. Discussion

Chest pain is one of the most common main symptoms for consultation, and chest pain can have many causes, including cardiovascular diseases, respiratory diseases, and even intestinal diseases [18]. In the present study, we found that in the differential diagnosis of AD and CAD, FLUTHE, a joint indicator formed by sFe, LDL, UA, TF, HDL, and eGFR, has a slight diagnostic superiority within 72 h of the symptom's onset. Over 72 h of the symptom's onset, FLUTHE's diagnostic power was slightly decreased, while the specificity was still satisfying.

Biomarkers have been investigated to prediagnose AD. D-dimer, a fibrinogen degradation product, has been widely used in clinical work and found to be an ideal predictor in early diagnosis [11]. However, it showed a high sensitivity in the first 24 h, but it declined over time [7, 19]. In our present study, a similar scenario was observed that the diagnostic efficacy of D-dimer within 24 h of onset was acceptable, but as the onset time increased, the content of D-dimer in the serum gradually decreased, and the diagnostic efficacy was also significantly reduced. Therefore, it is urgent to find more stable biomarkers for the differential diagnosis of AD and CAD.

Trace elements are usually relatively stable in the body, but changes in their content may lead to the occurrence of diseases. Iron, as one of the essential trace elements for the human body, has received extensive attention for a long time [20]. Recently, Li et al. demonstrated that iron deficiency contributes to the development of medial degeneration of the aorta in hypertensive patients [21]. In contrast, Edvinsson et al. revealed that compared with normal controls, Cu^2+^ and Zn^2+^ decreased and Fe^2+^ tended to increase in aortic tissue [17]. However, whether biomarkers of iron metabolism can be used as indicators for the diagnosis of AD is not yet known. We found that compared with CAD patients, the serum contents of TF and sFe were reduced in AD patients, but comparable levels of hemoglobin were observed in the blood of patients with AD and the control group. This may be due to the body giving priority to the production of hemoglobin when using iron [22, 23]. As shown in Figure 1, the contents of TF and sFe were stable in different time intervals, which indicated that TF and sFe may serve as stable diagnostic biomarkers. Unexpectedly, both TF and sFe are mediocre in the differential diagnosis of AD and CAD. These studies indicated that there is a paradox between serum iron and iron content in aortic tissue during the development of AD. Further in-depth research is needed to clarify the underlying reasons behind the emergence of this paradox.

To increase diagnostic performance, other biomarkers were enrolled by logistic regression, including liver function, kidney function, and blood biochemical indexes. Our results demonstrated that our model FLUTHE showed potential in both with 72 h and over 72 h for AD diagnosis while performed better over 72 h. Furthermore, so as for clinical practice, FLUTHE was transformed to a diagnostic score system, which is still valuable in AD diagnosis, as expected.

Lipoproteins, including HDL and LDL, have been widely investigated, especially in atherosclerosis [24]. Atherosclerosis is the main pathological alternation of CAD induced by imbalanced lipid metabolism and a maladaptive immune response inflicting chronic inflammation of the arterial wall [25]. Usually, in the serum of untreated patients with CAD, the level of LDL is significantly increased, while HDL may be reduced [26]. Similarly, it has been shown that imbalanced lipid metabolism also exists in AD, such as a higher level of soluble lectin-like oxidized LDL receptor-1 [27], and it has also been reported that atherosclerosis is a risk factor for AD [28]. In this study, the levels of LDL and HDL in patients with AD were higher than those in CAD patients. The use of lipid-lowering drugs may be the cause of lower LDL in patients with CAD than in patients with AD. Moreover, LDL and HDL can significantly improve the effectiveness of TF and sFe in the differential diagnosis of AD and CAD. This may also imply that for CAD patients who have not used lipid-lowering drugs, the differential diagnosis effectiveness of FLUTHE may be compromised.

UA is a vital biomolecule and the ultimate metabolite of purine in the human body [29]. Reports showed that the level of serum UA was associated with cardiovascular disease, stroke, and mortality in a J-curve manner [30, 31]. Recent studies also showed that higher serum levels of UA were related to a higher risk of AD and higher AD-related death rate [30, 32]. These results indicated that higher serum UA was associated with a higher incidence and mortality of AD. However, it is still poorly understood how serum UA increases the risk of AD. For CAD, it remains controversial how UA affects CAD in epidemiology [33, 34]. In our research, we found that serum UA was lower in AD than in CAD patients and that UA could increase the diagnostic performance in discriminating AD from CAD, while both levels of serum UA were within the normal range.

Finally, eGFR is used in early renal function assessment or late chronic renal function to assess disease progression and the degree of nephron loss [35]. A previous study indicated that for patients with type A AD stratified by different levels of eGFR, those with severe or moderate eGFR had a higher in-hospital mortality [36]. However, there is no further research on the different levels of eGFR in patients with AD and CAD. In our study, the level of eGFR in patients with AD was lower than that in CAD patients, which may be a result of false lumen formation in AD patients splitting peripheral blood flow and leading to acute ischemia in the renal artery.

Our study design divided the population into two different sets (a discovery set and a validation set), which suggested a convincing external extensibility of our model. Nevertheless, the sample size included is still not large enough, and a group for contemporary comparison is needed in future studies. The examinations to obtain the biomarker levels included in FLUTHE are routine and accessible for patients and can be easily performed in most hospitals. FLUTHE was found to be a preferable biomarker to differentiate AD from CAD than D-dimer, especially for patients more than 72 h past symptom onset. However, our study was also limited by its retrospective design, which inevitably resulted in admission bias, unobserved confounding factors, and missing values in our data. In addition, pulmonary embolism also has clinical symptoms similar to AD and CAD; however, our center rarely receives pulmonary embolism patients, and, therefore, we cannot obtain enough sample data. Hence, pulmonary embolism patients were not included in this study.

## 5. Conclusion

Taken together, our findings demonstrated that TF and sFe were relatively stable biomarkers in serum, and the combination of TF and sFe together with UA, eGFR, LDL, and HDL (namely, FLUTHE) is a promising indicator for discriminating AD from CAD, especially for patients with chest or back pain for more than 72 h.

## Figures and Tables

**Table 1:** Overall study design. The population was divided by the time admitted to the hospital. A total of 521 patients were recruited from three hospitals between Oct. 8, 2020, and Mar. 1, 2021. Patients admitted before Jan. 8, 2021, were divided into discovery set, and patients admitted after Jan.8, 2021, were divided into validation set. AD: aortic dissection; CAD: coronary artery disease; OCD: other cardiovascular diseases.

Study set	Discovery set			Validation set		
Study populations	*N* = 330 (162 AD + 135 CAD + 33 OCDs)			*N* = 191 (105 AD + 86 non − AD)		
	AD vs. non-AD	AD	non-AD	AD vs. non-AD	AD	non-AD
		162 (49.1%)	168 (50.9%)		105 (55.0%)	86 (45.0%)
	AD vs. CAD	AD	non-AD	AD vs. CAD	AD	non-AD
		162 (49.1%)	135 (40.9%)		105 (55.0%)	60 (31.4%)

**Table 2:** Distributions of TF and sFe of all patients with AD in different genders.

AD	Total patients	Male patients			Female patients		
		Total	Deficiency	Normal	Total	Deficiency	Normal
TF	267	200	110	90	67	39	28
sFe	267	200	115	85	67	19	48

AD: aortic dissection; sFe: serum iron; TF: transferrin.

**Table 3:** Diagnostic performance of FLUTHE in patients with AD vs. CAD in discovery set and validation set in different time manners.

Time manners	Sen	Spe	Accuracy	PPV	NPV	PLR	NLR
Discovery set							
≤72 h	0.723	0.952	0.803	0.986	0.417	15.06	0.29
>72 h	0.652	0.905	0.837	0.714	0.877	6.86	0.38
Validation set							
≤72 h	0.765	0.813	0.776	0.928	0.52	4.07	0.29
>72 h	0.954	0.905	0.931	0.913	0.92	10.04	0.05

AD: aortic dissection; CAD: coronary artery disease; NLR: negative likelihood ratio; NPV: negative predictive value; PLR: positive likelihood ratio; PPV: positive predict value; Sen: sensitivity; Spe: specificity; FLUTHE: prediction index including TF, sFe, LDL, HDL, uric acid, and eGFR. Optimal threshold value was obtained from the data, which was the threshold leading to the maximum summation of sensitivity and specificity (i.e., the Youden index).

**Table 4:** Distributions of different possibility according to diagnostic score system and OR value compared with low possibility group.

Possibility	Number of patients		OR	95% CI	
	ALL	AD	Compared with low possibility	Lower	Upper
Low (score 0-3)	40	4			
Medium (score 4-5)	154	70	7.50	2.54	22.10
High (score 6-10)	211	188	73.57	24.00	225.48

AD: aortic dissection; OR: odds ratio.

## Data Availability

The data that support the findings of this study are available from the corresponding authors upon reasonable request.

## Abbreviations

AD: Aortic dissection
CAD: Coronary artery disease
OCDs: Other cardiovascular diseases
CT: Computed tomography
CTA: CT angiography
MRI: Magnetic resonance imaging
MRA: MRI angiography
sFe: Serum iron
TF: Transferrin
TIBC: Total iron-binding capacity
TFS: Transferrin saturation
UIBC: Unsaturated iron-binding capacity
sTFR: Soluble transferrin receptor
UA: Uric acid
eGFR: Estimated glomerular filtration rate
LDL: Low-density lipoprotein
HDL: High-density lipoprotein.

## Supplementary Materials

Supplementary Materials Supplemental Figure S1: study design and objectives in the discovery and validation set. Supplemental Table S1: baseline characteristics of patients with AD vs. CAD and OCDs in discovery set. Supplemental Table S2: baseline characteristics of patients with AD vs. non-AD diseases in validation set. Supplemental Table S3: logistic regression analysis of sFe, TF, LDL, HDL, eGFR, and UA in discovery set. Supplemental Table S4: diagnostic performance of FLUTHE at threshold value of 0.648 in discovery set and validation set. Supplemental Table S5: diagnostic performance of FLUTHE in patients with AD vs. non-AD in discovery set and validation set in different time intervals.
