# Supplementary material for: A Novel Serum Biomarker Model to Discriminate Aortic Dissection from Coronary Artery Disease
Source: Dis Markers. 2022 Jul 20;2022:9716424. doi: 10.1155/2022/9716424 (PMC9329023; doi:10.1155/2022/9716424)
Supplement: Supplementary Materials — Supplemental Figure S1: study design and objectives in the discovery and validation set. Supplemental Table S1: baseline characteristics of patients with AD vs. CAD and OCDs in discovery set. Supplemental Table S2: baseline characteristics of patients with AD vs. non-AD diseases in validation set. Supplemental Table S3: logistic regression analysis of sFe, TF, LDL, HDL, eGFR, and UA in discovery set. Supplemental Table S4: diagnostic performance of FLUTHE at threshold value of 0.648 in discovery set and validation set. Supplemental Table S5: diagnostic performance of FLUTHE in patients with AD vs. non-AD in discovery set and validation set in different time intervals. [file 9716424.f1.docx]

**Supplemental Materials**

**A novel serum biomarker model to discriminate aortic dissection from coronary artery disease**

Peijiang Lu^1*^, Xin Feng^1*^, Rui Li^1*^, Peng Deng^1^, Shiliang Li^1^, Jiewen Xiao^1^, Jing Fang^1,2^, Xingyu Wang^1^, Chang Liu^3^, Qiuxia Zhu^3^, Jing Wang^1^, Zemin Fang^1^, Lu Gao^4^, Sen Guo^4^, Xue-Jun Jiang^3^, Xue-Hai Zhu^1,2^, Tingting Qin^5^, Xiang Wei^1,2#^, Xin Yi^3#^, Ding-Sheng Jiang^1,2#^

^1^Division of Cardiothoracic and Vascular Surgery, Sino-Swiss Heart-Lung Transplantation Institute, Tongji Hospital, Tongji Medical College, Huazhong University of Science and Technology, Wuhan, Hubei, China; ^2^Key Laboratory of Organ Transplantation, Ministry of Education; NHC Key Laboratory of Organ Transplantation; Key Laboratory of Organ Transplantation, Chinese Academy of Medical Sciences, Wuhan, Hubei, China; ^3^Department of Cardiology, Renmin Hospital of Wuhan University, Wuhan, Hubei, China; ^4^Department of Cardiology, the First Affiliated Hospital of Zhengzhou University, Zhengzhou, Henan, China; ^5^Department of Biliary-Pancreatic Surgery, Tongji Hospital, Tongji Medical College, Huazhong University of Science and Technology, Wuhan, Hubei, China.

***These authors contribute equally to this work**

**Correspondence to**

**Ding-Sheng Jiang, MD**

Division of Cardiothoracic and Vascular Surgery

Tongji Hospital, Tongji Medical College

Huazhong University of Science and Technology

1095 Jiefang Ave., Wuhan 430030, China

Tel/Fax: 86-27-6937-8454; E-mail: [jds@hust.edu.cn](mailto:jds@hust.edu.cn)

**Or**

**Xin Yi, MD**

Department of Cardiology, Renmin Hospital of Wuhan University

Cardiovascular Research Institute, Wuhan University

Jiefang Rd 238, Wuhan 430060, China

Tel/Fax: 86-27-8804-1911; E-mail: [yixin321624@126.com](mailto:yixin321624@126.com)

**Or**

**Xiang Wei, MD**

Division of Cardiothoracic and Vascular Surgery

Tongji Hospital, Tongji Medical College

Huazhong University of Science and Technology

1095 Jiefang Ave., Wuhan 430030, China

Tel/Fax: 86-27-6937-8454; E-mail: [xiangwei@tjh.tjmu.edu.cn](mailto:xiangwei@tjh.tjmu.edu.cn)

**Supplemental Methods.**

- 1. **Study Samples**

We enrolled the patients diagnosed with aortic dissection, coronary artery disease, or other cardiovascular diseases according to the diagnosis criteria as mentioned in one of the three hospitals from Oct.8, 2020-Mar.1, 2021, and the patients should include complete clinical information, including iron metabolism-related biomarkers, blood routine, blood chemistry, liver and kidney function tests, D-dimer, CT/CTA, MRI/MRA, and ECG.

Patients were diagnosed with AD if they had chest pain or stomachache together with CTA/MRA indicating dissection existing and then classified according to the Stanford system. Patients were diagnosed with CAD if they had chest pain together with (a) diagnostic serial ECG changes such as new pathological Q waves or ST-segment and T-wave changes and (b) coronary angiogram indicating pathological changes in coronary arteries and a serum creatine kinase-myocardial band elevation more than twice the normal level or cardiac troponin I (cTnI) level >100 μg/ml.

The diagnosis of OCDs was confirmed by ultrasound cardiogram and CT. Patients diagnosed with CAD or OCDs were regarded as non-AD

- 1. **Measurements of Iron Metabolism and Other Markers**

Biomarkers of iron metabolism tests were performed in Tongji Hospital, Huazhong University of Science and Technology, Wuhan, China. Blood samples from participants were collected after they entered the hospital and before surgery and were then sent to the clinical laboratory of Tongji Hospital for tests. The same procedures were handled for different case-control groups. The serum levels of sFe, ferritin, TF, sTFR, and UIBC were tested by a fully automated analyzer (Roche/Hitachi Cobas c 701/702, Roche Diagnostics GmbH) using validated laboratory methods at admission. TFS and TIBC were calculated simultaneously by the analyzer. All kits needed in the tests were purchased from Roche Diagnostics.

A D-dimer test was generally ordered for patients with suspicion of AD, according to the recommendations of ESC 2014, to investigate the diagnostic performance of the novel module[1]. All patients enrolled in the study finally had confirmatory medical imaging examination regardless of the result of the D-dimer test. D-dimer was detected using an STA-R MAX coagulation analyzer and original reagents (Diagnostica Stago, Saint-Denis, France). The upper limit was 21.00 μg/mL. Patients with D-dimer over 21.00 μg/mL were regarded as 21.00 μg/mL.

Other markers were all collected from Baseline characteristics and surgical information of patients were collected from medical records and confirmed by the study doctors. Other tests are done independently by each hospital.

- 1. **Statistical Analysis**

The diagnostic performance of biomarkers of iron metabolism for distinguishing AD from all other diseases, CAD or OCDs, was assessed using nonparametric receiver operating characteristic curve (ROC) analysis. The area under the ROC curve (AUROC), sensitivity, specificity, accuracy, and 2 likelihood ratios were suggested for positive and negative test results, and positive predictive value and negative predictive value were calculated. The threshold value with the highest Youden index was defined as the cutoff value. The model performance was evaluated by the area under the curve (AUC) from cross-validation analysis. Analyses were performed by SPSS (version 22.0) software. Figures were made by the R ggplot2 package (version 4.0.3) and GraphPad (version 9.0). All *P* values were 2-tailed and were not adjusted for multiple testing, and *P* <0.05 was considered statistically significant. Missing data were deleted in further analysis using list deletion.

**Supplemental Figure S1. Study Design and Objectives in the Discovery and Validation Set.** 521 patients were collected from three hospitals from Oct. 1, 2021 to Mar. 1, 2021.Patients admitted before Jan 8. 2021 were assigned to discovery set and others were validation set. AD indicates aortic dissection; CAD indicates coronary artery disease; OCDs indicates other cardiovascular diseases.

**
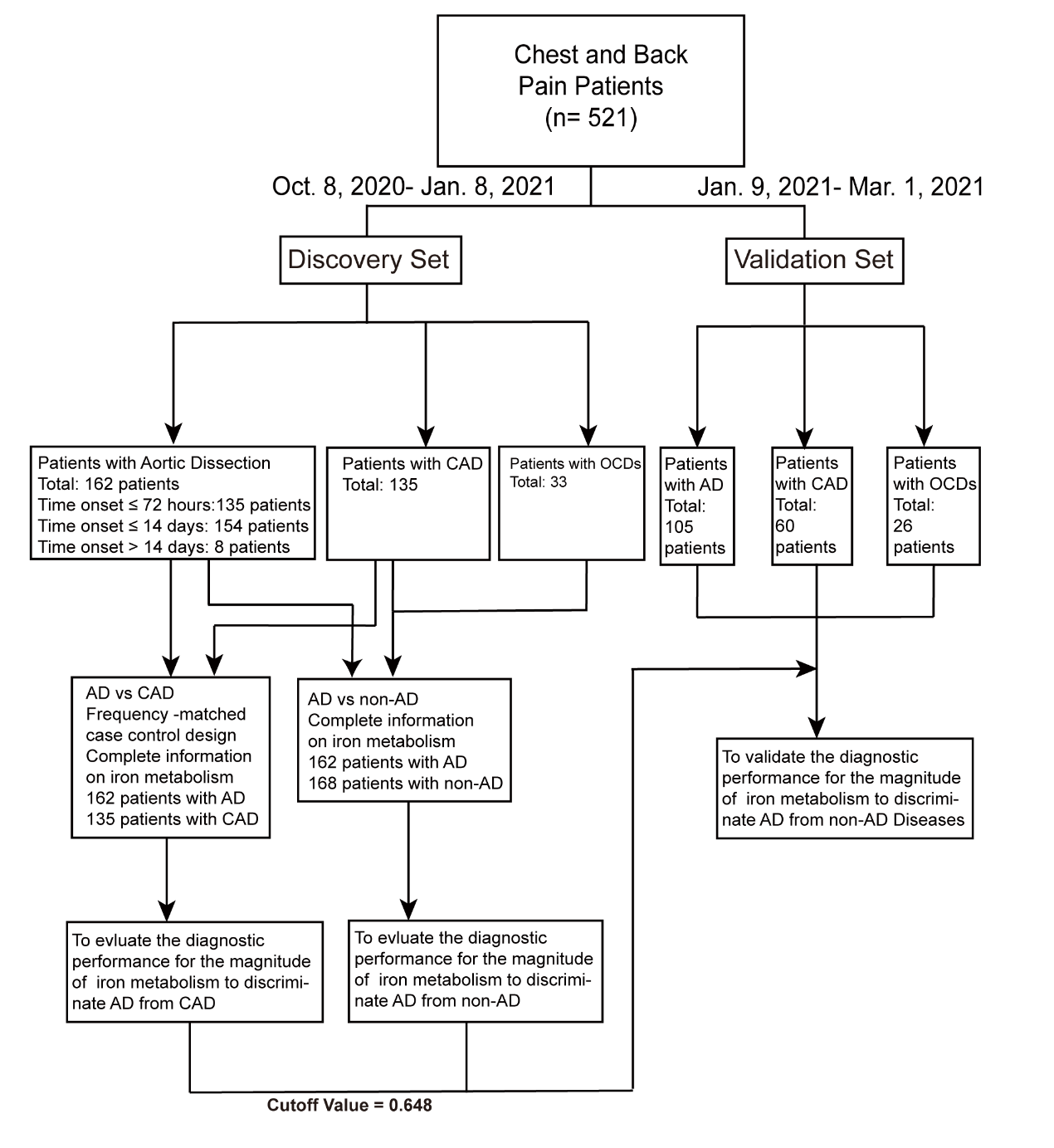
**

**Supplemental Table S1. Baseline Characteristics of Patients with AD vs. CAD and OCDs in Discovery Set.**

|  | Aortic Dissection | | Coronary Artery Disease | | *P* value | Other Cardiovascular Diseases | | *P* value |  |
| --- | --- | --- | --- | --- | --- | --- | --- | --- | --- |
|  | No. | Mean (95% CI or %) | No. | Mean (95% CI or %) |  | No. | Mean (95% CI or %) |  |  |
|  |  |  |  |  |  |  |  |  |  |
|  |  |  |  |  |  |  |  |  |  |
| Age (years) | 162 | 56.82 (54.74, 58.97) | 135 | 59.46 (57.81, 62.82) | 0.062 | 33 | 62.50 (57.13, 68.14) | 0.084 |  |
| Gender (male) | 162 | 122 (75.3%) | 135 | 74 (54.8%) | **<0.0001** | 33 | 18 (54.5%) | **0.016** |  |
| Smoking (current) | 162 | 84 (52.1%) | 135 | 61 (45.2%) | 0.321 | 33 | 17 (51.52%) | 0.972 |  |
| Diabetes | 162 | 55 (31.8%) | 135 | 38 (26.4%) | 0.057 | 33 | 13 (39.39%) | 0.145 |  |
| Hypertension | 162 | 117 (72.5%) | 135 | 54 (40.0%) | **<0.0001** | 33 | 11 (33.3%) | **<0.0001** |  |
| Lipid-lowering Drugs | 162 | 82 (50.7%) | 135 | 135(100%) | **<0.0001** | 33 | 10 (30.4%) | 0.037 |  |
| Ferritin (μg/mL) | 162 | 318.54 (273.17, 376.60) | 135 | 307.70 (253.09, 374.43) | 0.796 | 33 | 301.36 (185.22, 472.67) | 0.344 |  |
| sFe (μmol/mL) | 162 | 11.67 (10.48, 12.67) | 135 | 15.11 (12.90, 15.44) | **0.031** | 33 | 12.97 (10.86, 15.41) | 0.874 |  |
| UIBC (μmol/mL) | 162 | 35.85 (34.20, 37.47) | 133 | 36.25 (34.59, 38.11) | 0.737 | 33 | 35.38 (32.05, 38.78) | 0.309 |  |
| TIBC (μmol/mL) | 162 | 47.58 (46.21, 49.02) | 133 | 50.79 (49.30, 52.44) | **0.003** | 33 | 48.34 (44.89, 51.88) | 0.203 |  |
| TF (g/L) | 162 | 2.07 (2.01, 2.14) | 135 | 2.27 (2.21, 2.34) | **<0.0001** | 33 | 2.10 (1.93, 2.27) | 0.819 |  |
| TFS (%) | 162 | 24.42 (22.21, 27.19) | 116 | 26.91 (22.82, 31.60) | 0.054 | 33 | 28.04 (25.47, 30.60) | 0.622 |  |
| sTFR (mg/mL) | 158 | 2.88 (2.68, 3.14) | 115 | 3.08 (2.77, 3.51) | 0.944 | 33 | 3.11 (2.72, 3.59) | 0.16 |  |
| D-dimer (μg/mL) | 116 | 4.80 (1.37, 14.56) | 90 | 0.27 (0.17, 0.53) | **<0.0001** | 33 | 0.19 (0.12, 0.62) | **<0.0001** |  |
| TnI (μg/mL) | 148 | 957.50 (182.31, 2022.90) | 101 | 510.75 (9.59, 1622.51) | 0.517 | 33 | 282.48 (6.37, 909.74) | 0.226 |  |
| eGFR (ml/min/1.73m^2） | 162 | 80.35 (76.20, 84.12) | 87 | 112.92 (106.08, 119.36) | **<0.0001** | 33 | 96.20 (82.61, 110.13) | 0.548 |  |
| UA (μmol/L) | 162 | 347.21 (328.47, 365.24) | 87 | 374.51 (354.47, 395.24) | **0.043** | 33 | 365.19 (313.20, 411.35) | **0.03** |  |
| LDL (mmol/mL) | 145 | 2.68 (2.53, 2.83) | 98 | 2.36 (2.18, 2.54) | **0.006** | 25 | 2.17 (1.84, 2.56) | **0.007** |  |
| HDL (mmol/mL) | 145 | 1.08 (1.04, 1.13) | 98 | 0.97 (0.93, 1.02) | **0.003** | 25 | 1.01 (0.90, 1.13) | 0.451 |  |

HDL indicates high-density lipoprotein; LDL indicates low-density lipoprotein; UA indicates uric acid; eGFR indicates estimated glomerular filtration rate; TnI indicates cardiac troponin I; sFe indicates serum iron; TF indicates transferrin; TIBC indicates total iron binding capacity; UIBC indicates unsaturated iron-binding capacity; sTFR indicates soluble transferrin receptor; TFS indicates transferrin saturation.

**Supplemental Table S2. Baseline Characteristics of Patients with AD vs. non-AD Diseases in Validation Set.**

|  | Aortic Dissection | | non-AD diseases | | *P* value |
| --- | --- | --- | --- | --- | --- |
|  | No. | Mean (95% CI or %) | No. | Mean (95% CI or %) |  |
| Age (years) | 105 | 60.00 (51.27, 69.87) | 86 | 62.50 (57.13, 68.14) | 0.088 |
| Gender (male) | 105 | 78 (74.3%) | 86 | 50 (58.1%) | **0.042** |
| Smoking (current) | 105 | 64 (60.7%) | 86 | 7 (8.2%) | **0.01** |
| Diabetes | 105 | 42 (40.0%) | 86 | 25 (29.0%) | 0.115 |
| Hypertension | 105 | 38 (36.2%) | 86 | 27 (31.4%) | 0.071 |
| Lipid-lowering Drugs | 105 | 42 (40.0%) | 86 | 81 (94.2%) | **<0.0001** |
| Ferritin (ug/mL) | 105 | 762.44 (381.372, 1367.40) | 86 | 479.47 (221.22, 965.53) | 0.174 |
| sFe (μmol/mL) | 105 | 9.88 (8.86, 11.07) | 86 | 10.74 (9.18, 12.29) | 0.370 |
| UIBC (μmol/mL) | 105 | 38.09 (34.58, 42.78) | 86 | 35.71 (33.34, 38.20) | 0.402 |
| TIBC (μmol/mL) | 105 | 45.76 (43.77, 47.63) | 86 | 46.46 (44.37, 48.52) | 0.625 |
| TF (g/mL) | 105 | 1.81 (1.73, 1.88) | 86 | 2.08 (1.98, 2.18) | **0.0009** |
| TFS (%) | 105 | 23.91 (21.10, 26.73) | 63 | 27.55 (24.08, 31.10） | 0.115 |
| sTFR (mg/mL) | 105 | 3.27 (2.99, 3.61) | 86 | 3.87 (3.42, 4.43) | 0.059 |
| D-dimer (μg/mL) | 105 | 7.12 (5.44, 8.91) | 86 | 1.02 (0.72, 1.36) | **0.001** |
| TnI (μg/mL) | 95 | 1047.18 (227.47, 2238.73) | 83 | 2614 (435.22, 2238.73) | 0.396 |
| eGFR (ml/min/1.73m^2） | 105 | 77.25 (72.53, 82.41) | 86 | 83.86 (75.68, 91.68) | 0.147 |
| UA (μmol/L) | 105 | 359.67 (337.88, 382.34) | 86 | 392.71 (363.19, 426.72) | 0.145 |
| LDL (mmol/mL) | 95 | 2.62 (2.47,2.80) | 86 | 1.84 (1.68, 2.01) | **<0.0001** |
| HDL (mmol/mL) | 95 | 1.08 (1.02, 1.140) | 86 | 0.93 (0.88, 0.98) | **<0.0001** |

HDL indicates high-density lipoprotein; LDL indicates low-density lipoprotein; UA indicates uric acid; eGFR indicates estimated glomerular filtration rate; TnI indicates cardiac troponin I; sFe indicates serum iron; TF indicates transferrin; TIBC indicates total iron binding capacity; UIBC indicates unsaturated iron-binding capacity; sTFR indicates soluble transferrin receptor; TFS indicates transferrin saturation.

**Supplemental Table S3. Logistic Regression Analysis of sFe, TF, LDL, HDL, eGFR, UA in Discovery Set.**

|  | β Coefficient | S.E. | Wald | df | significance | OR | 95% CI | | β Coefficient  (Standardized) | Score |
| --- | --- | --- | --- | --- | --- | --- | --- | --- | --- | --- |
|  |  |  |  |  |  |  | lower | upper |  |  |
| HDL | 1.674 | 0.644 | 6.751 | 1 | **0.009** | 5.333 | 1.51 | 18.9 | 0.470 | 2 |
| LDL | 0.854 | 0.226 | 14.354 | 1 | **<0.0001** | 2.350 | 1.51 | 3.66 | 0.776 | 2 |
| UA | -0.003 | 0.001 | 5.278 | 1 | **0.022** | 0.997 | 0.99 | 1 | -0.409 | 2 |
| eGFR | -0.037 | 0.007 | 30.739 | 1 | **<0.0001** | 0.964 | 0.95 | 0.98 | -1.217 | 4 |
| sFe | -0.067 | 0.024 | 7.675 | 1 | **0.006** | 0.935 | 0.89 | 0.98 | -0.490 | 2 |
| TF | -1.382 | 0.445 | 9.652 | 1 | **0.002** | 0.251 | 0.11 | 0.6 | -0.592 | 4 |
| C | 4.831 | 1.471 | 10.78 | 1 | **0.001** |  |  |  |  |  |

HDL indicates high-density lipoprotein; LDL indicates low-density lipoprotein; UA indicates uric acid; eGFR indicates estimated glomerular filtration rate; sFe indicates serum iron; TF indicates transferrin.

**Supplemental Table S4.** **Diagnostic Performance of FLUTHE at threshold value of 0.648 in Discovery Set and Validation Set.**

| **Comparison** | **Sensitivity** | **Specificity** | **Accuracy** | **PPV** | **NPV** | **PLR** | **NLR** |
| --- | --- | --- | --- | --- | --- | --- | --- |
| **Discovery Set** |  |  |  |  |  |  |  |
| **AD Versus CAD** | 0.713 | 0.917 | 0.793 | 0.926 | 0.682 | 8.55 | 0.31 |
| **AD Versus non-AD** | 0.713 | 0.854 | 0.782 | 0.846 | 0.723 | 4.86 | 0.34 |
| **Validation Set** |  |  |  |  |  |  |  |
| **AD Versus CAD** | 0.802 | 0.833 | 0.812 | 0.913 | 0.667 | 4.82 | 0.22 |
| **AD Versus non-AD** | 0.802 | 0.778 | 0.767 | 0.793 | 0.731 | 2.94 | 0.25 |

AD indicates aortic dissection; CAD indicates coronary artery disease; NLR, negative likelihood ratio; NPV, negative predictive value; PLR positive likelihood ratio; PPV, positive predict value; FLUTHE indicates prediction index including TF, sFe, LDL, HDL, Uric Acid, eGFR. Optimal threshold value obtained from the data, which was the threshold leading to the maximum summation of sensitivity and specificity (ie, the Youden index).

**Supplemental Table S5.** **Diagnostic Performance of FLUTHE in Patients with AD vs. non-AD in Discovery Set and Validation Set in Different Time Intervals.**

| **Comparison** | **Time Manners** | **Threshold Value** | **Sensitivity** | **Specificity** | **Accuracy** | **PPV** | **NPV** | **PLR** | **NLR** |
| --- | --- | --- | --- | --- | --- | --- | --- | --- | --- |
| **Discovery Set** |  |  |  |  |  |  |  |  |  |
| **FLUTHE** | ≤72h | 0.648 | 0.723 | 0.884 | 0.750 | 0.961 | 0.442 | 6.16 | 0.32 |
| **FLUTHE** | >72h | 0.648 | 0.652 | 0.843 | 0.821 | 0.534 | 0.897 | 4.15 | 0.41 |
| **Validation Set** |  |  |  |  |  |  |  |  |  |
| **FLUTHE** | ≤72h | 0.648 | 0.765 | 0.769 | 0.752 | 0.793 | 0.731 | 2.94 | 0.25 |
| **FLUTHE** | >72h | 0.648 | 0.954 | 0.786 | 0.860 | 0.778 | 0.957 | 4.45 | 0.06 |

AD indicates aortic dissection; NLR, negative likelihood ratio; NPV, negative predictive value; PLR positive likelihood ratio; PPV, positive predict value; FLUTHE indicates prediction index including TF, sFe, LDL, HDL, Uric Acid, eGFR. Optimal threshold value obtained from the data, which was the threshold leading to the maximum summation of sensitivity and specificity (ie, the Youden index).

**References**

1. Erbel R., Aboyans V., Boileau C., Bossone E., Bartolomeo R. D., Eggebrecht H., et al. 2014 ESC Guidelines on the diagnosis and treatment of aortic diseases: Document covering acute and chronic aortic diseases of the thoracic and abdominal aorta of the adult. The Task Force for the Diagnosis and Treatment of Aortic Diseases of the European Society of Cardiology (ESC). Eur Heart J. 2014;35(41):2873-926.
